# Targeting Fatty Acids in Liver Cancer: Molecular Insights and Drug Approaches

**DOI:** 10.3390/biom16020329

**Published:** 2026-02-20

**Authors:** Antonio Cigliano, Dora Pischedda, Claudio Pandino, Grazia Galleri, Diego F. Calvisi

**Affiliations:** 1 Faculty of Medicine, Saint Camillus International University of Health Sciences, 00131 Rome, Italy; 2 Department of Medicine, Surgery, and Pharmacy, University of Sassari, 07100 Sassari, Italy; d.pischedda@studenti.uniss.it; 3 Department of Biomedical Sciences, University of Sassari, 07100 Sassari, Italy; claudio.pandino96@gmail.com (C.P.); galleri@uniss.it (G.G.)

**Keywords:** primary liver cancer, lipid metabolism, metabolic reprogramming, tumor microenvironment, drug resistance, personalized medicine, innovative therapies

## Abstract

Primary liver cancer (PLC), commonly classified as hepatocellular carcinoma (HCC) and intrahepatic cholangiocarcinoma (ICC), is a highly aggressive malignancy with a dismal prognosis. Recent research has highlighted the crucial role of dysregulation of fatty acid metabolism in HCC progression and therapeutic resistance. Here, with a focus primarily on HCC, we review how alterations in the processes involving fatty acids dynamically contribute to the survival, proliferation, and development of the drug resistance of PLC cells. In particular, increased expression of fatty acid transporters, reprogramming of de novo lipogenesis, and altered fatty acid oxidation trigger the upregulation of oncogenic signaling pathways and adaptation to nutrient-deprived conditions inducing the rapid proliferation of PLC cells. Furthermore, fatty acid metabolism influences immune cell function and angiogenesis, thereby shaping the tumor microenvironment and promoting the progression of PLC. This review explores the complex relationship between fatty acid metabolism and the progression of PLC. It discusses future directions regarding the most promising druggable targets and their current status in clinical trials. Furthermore, it examines the advancement of innovative therapeutic strategies and highlights the significant challenges in targeting fatty acid metabolism. Finally, it discusses how precision therapies focused on fatty acid metabolism can be effectively integrated with current treatments.

## 1. Introduction

**Figure 1:**
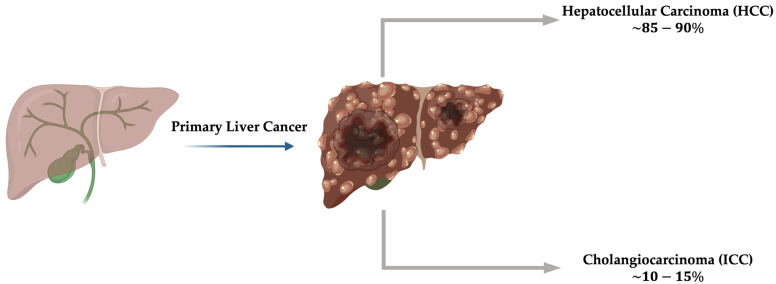
Primary liver cancers, including HCC (85–90%) and ICC (10–15%), arising from the malignant transformation of hepatocytes and cholangiocytes. This image was created using the BioRender online tool (www.biorender.com, accessed on 10 November 2025).

Primary liver cancer (PLC) ranks as the third (second in males) most common cause of cancer-related death worldwide, with approximately 900,000 people being diagnosed each year and its incidence steadily increasing [1]. The Global Cancer Observatory (GCO) (https://gco.iarc.who.int accessed on 18 December 2025) estimates that, by 2045, about 1.5 million people will be affected by PLC, posing a serious health problem partly due to a constant increase in the incidence of metabolic risk factors, including, among others, excess body weight, diabetes, and alcohol consumption [1]. PLCs include mostly hepatocellular carcinoma (HCC), accounting for up to 90% of all cases, and intrahepatic cholangiocarcinoma (ICC), accounting for the remaining 10–15% [2,3]. Chronic HBV and HCV infections are among the most common risk factors in PLC development. However, the frequency of these infections has decreased in recent years due to the development of effective antiviral drugs and the implementation of successful vaccination programs [4]. Other common risk factors comprise alcohol abuse, metabolic dysfunction-associated steatotic liver disease (MASLD) that includes obesity and diabetes, aflatoxin exposure for HCC, and inflammation of the biliary tract for ICC caused by primary sclerosing cholangitis (PSC), cholestasis, and liver fluke infestation [2,3,5,6]. Nevertheless, PLCs exhibit a complex heterogeneity, making their early diagnosis and development of novel therapies challenging (Figure 1).

Reprogramming cellular metabolism is a well-established hallmark of cancer [7]. The liver is a metabolic organ that plays a central role in all metabolic processes [8]. The risk factors associated with PLCs’ development and progression usually induce metabolic alterations and rearrangements. Characterizing these metabolic alterations during liver carcinogenesis would have significant implications, from identifying pathogenic mechanisms and therapeutic targets to discovering novel prognostic biomarkers. Metabolic dysfunction supports the survival of cancer cells in a hostile microenvironment, and altered energy metabolism provides the cell with the flexibility to thrive in the environment, adapting to the availability of different energy sources. Cancer cells use numerous nutritional sources to promote tumor progression, including glucose, tricarboxylic acid cycle intermediates, amino acids, nucleotides, and lipids [9]. Typically, PLC development follows chronic inflammation, often secondary to viral hepatitis, alcoholic liver disease, and MASLD, creating a tumor-permissive microenvironment that facilitates malignant transformation. This chronic inflammatory setting induces repetitive cycles of hepatocyte damage and regeneration, determining genomic instability and the acquisition of oncogenic mutations in critical regulatory pathways, including Wnt/β-catenin, PI3K/Akt/mTOR, and RAS/RAF/MAPK signaling cascades. As these genetic aberrations accumulate, pre-neoplastic lesions progress to early-stage HCC, characterized by dysregulated cellular proliferation and the evasion of apoptosis. Concomitantly, the tumor microenvironment (TME) undergoes dramatic remodeling, activating hepatic stellate cells and promoting fibrosis and abnormal angiogenesis. This aberrant vascular network, lacking normal structural organization and functional integrity, not only supplies oxygen and nutrients to sustain tumor growth but also creates hypoxic niches that drive the expression of hypoxia-inducible factors (HIFs), further amplifying aggressive phenotypes through metabolic reprogramming. Numerous studies have described the pivotal role of lipid metabolism in the pathogenesis of PLC, which is a consequence of these biomolecules being involved in different biological processes, from energy storage and metabolism to signal transduction, cell–cell recognition, inflammation, and immunoregulation, but also epigenetic regulation. Indeed, lipids have emerged as critical factors that fuel tumor growth and contribute to cancer adaptation to therapeutic pressures [10,11,12,13]. Indeed, the metabolic plasticity exhibited by PLC cells represents a fundamental adaptive mechanism, with pronounced shifts toward aerobic glycolysis, glutaminolysis, and altered lipid metabolism, collectively enhancing cellular resilience while generating immunosuppressive metabolites that reshape the surrounding tissue architecture.

This review focuses on the evolving landscape of lipid metabolism and how its alteration impacts de novo lipogenesis (DNL) and fatty acid oxidation (FAO), leading to PLC progression and resistance to conventional treatments. Additionally, we will discuss the potential implications of targeting these metabolic pathways with novel targeted therapies and immunotherapies that may enhance treatment efficacy and improve patient outcomes.

## 2. Dysregulation of Lipid Metabolism in Primary Liver Cancer

**Figure 2:**
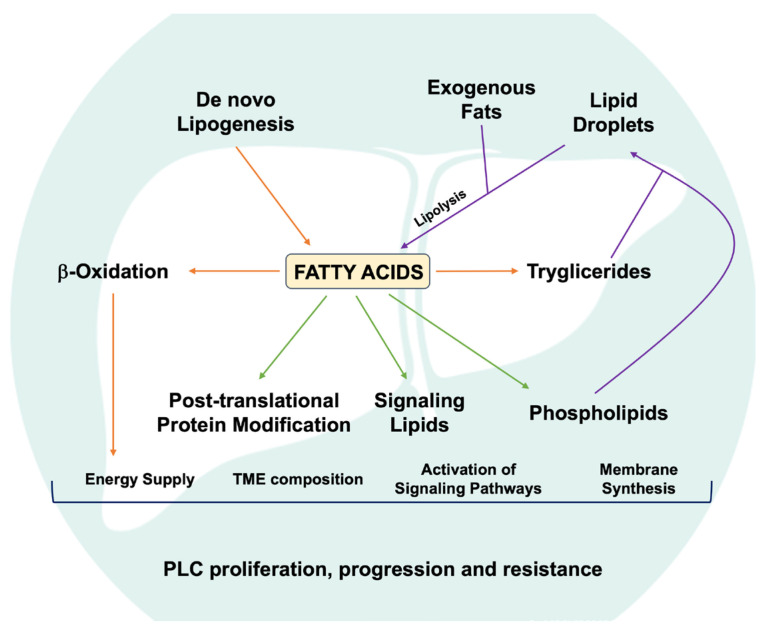
Alterations in fatty acid metabolism can promote diverse aspects of PLC development, affecting ability to proliferate, tumor progression and response to treatment. PLC, primary liver cancer; TME, tumor microenvironment. This image was modified from Wang M et al. doi: 10.2217/hep-2016-0012.

Cancer cells rewire energy production to sustain development and provide adaptation to survival, proliferation, invasion, and metastasis. Several studies have highlighted the importance of rewiring lipid metabolism for cancer cells, particularly the alterations in fatty acids (FAs) uptake and DNL that lead to tumor growth and therapeutic resistance [14]. On the other hand, dysregulated FAO also plays a crucial role; indeed, targeting such oxidation may be an effective anti-tumor approach, limiting energy availability and precursors to be used as building blocks [15,16]. Most cells cope with the need for FAs from exogenous dietary sources or DNL. Approximately 15 to 25% of all FAs originate from DNL, allowing the synthesis of FAs up to the D9 position, while other FAs need to be incorporated into the diet [17]. On the other hand, cholesterol is almost entirely (80%) synthesized by the cells and highly regulated, especially by the liver [18]. In cancer and normal cells, FAs and cholesterol are key biomolecules that exert various biological functions. They serve as biosynthetic precursors for energy storage, membrane lipids, first or second messengers in signal transduction and the molecular recognition process, and modifying groups added to proteins post-translationally [19,20,21] (Figure 2).

### 2.1. Altered Fatty Acid Uptake

**Figure 3:**
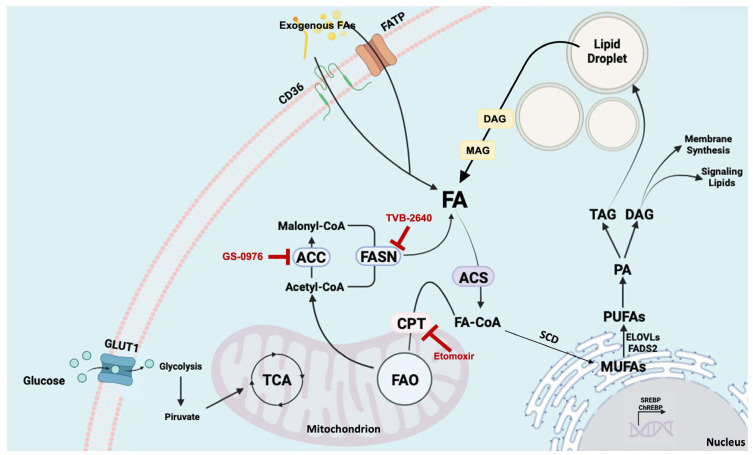
Overview of lipid metabolism with schematic anabolic and catabolic pathways. Exogenous FAs are transported into the cytoplasm via specialized transporters such as CD36 and FATPs. In cancer cells, FAs are also synthesized through de novo lipogenesis (DNL), which promotes the expression of DNL core enzymes by SREBP1 transcription factor. FAs and their products subsequently enter the mitochondria to produce NADPH and acetyl-CoA through β-oxidation for energy production to promote cell survival, metastasis, and chemoresistance. Several points of the lipogenic pathways can be targeted with specific inhibitors, some in clinical trials (indicated with blunted red arrows in the Figure). Abbreviations: ACC, acetyl-CoA carboxylase; ACS, acetyl-CoA synthase; CD36, cluster of differentiation 36; ChREBP, carbohydrate response element-binding protein; CPT, carnitine palmitoyl transferase; DAG, diacylglycerol; ELOVLs, elongation of very long-chain fatty acid protein; FA, fatty acid; FA-CoA, fatty acyl-CoA; FADS2, fatty acid desaturase 2; FAO, fatty acid oxidation; FASN, fatty acid synthase; FATP, fatty acid transport protein family; GLUT1, glucose transporter 1; MAG, monoacylglycerol; MUFA, monounsaturated fatty acid; PA, phosphatidic acid; PUFA, polyunsaturated fatty acid; SCD, stearoyl-CoA desaturase1; SREBP, sterol regulatory element-binding protein; TAG, triacylglycerol; TCA, tricarboxylic acid. This image was modified from Ward AV et al. doi: 10.1007/s10911-021-09505-3 using the BioRender online tool (www.biorender.com, accessed on 10 November 2025).

In HCC, as in many other types of cancer [22], the uptake of exogenous FAs is often significantly increased, providing a way to promote growth and proliferation (Figure 3). External FA uptake is an active mechanism [23] driven by the upregulation of the fatty acid translocase (FAT, also called CD36) on the cell membrane surface, fatty acid transport protein family (FATPs or SLC27 family) responsible for the transport of long-chain fatty acids, plasma membrane fatty acid-binding proteins (FABPs) that mediate intracellular trafficking and metabolism, and also by the activity of lipases that release them from circulating lipids [24]. The dysregulation of fatty acid uptake, particularly the overexpression of CD36, plays a significant role in the progression and therapeutic resistance of HCC. Given CD36′s crucial role in lipid metabolism, CD36 expression was analyzed across different human tissues in the Cancer Genome Atlas (TCGA) dataset. These analyses suggest that this protein is essential for growth, invasion, and metastasis. Indeed, several investigations have reported that CD36 expression is strictly linked to metastatic initiation and progression in different tumors, such as oral carcinoma, melanoma, breast cancer, and ovarian cancer. Moreover, studies on CD36 expression have correlated this protein with poor prognosis and increased tumor aggressiveness, indicating that HCC cells rely on exogenous free FAs to meet diverse metabolic needs, and highlighting the effect of CD36 knockdown, which negatively impacts cancer survival and progression [25].

CD36, a transmembrane glycoprotein expressed in various tissues, is a scavenger receptor involved in immune recognition, inflammation, molecular adhesion, apoptosis, and lipid uptake [26]. The abnormally high CD36 expression in HCC induces epithelial–mesenchymal transition (EMT) by activating the PI3K/AKT/mTOR pathway, promoting tumor metastasis [27,28]. Another route to promote EMT in HCC is that increased FA uptake induces an altered transcriptional activity of inflammation-related factors, such as NF-κB, AP-1, STAT3, and HIF-1α with the concomitant activation of Wnt and TGF-β oncogenic signaling pathways [27,28,29]. Additionally, CD36 regulates lipid metabolism, mediating the uptake of oxidized LDL (ox-LDL). Clearly, metabolic reprogramming affects different pathways, i.e., CD36-Nogo-B-YAP, through the altered expression of CEBPβ expression, ultimately leading to modified oxLDL metabolism triggering carcinogenetic signaling in MASLD (ex NAFLD)-associated HCCs [30]. In the HCC tumor microenvironment (TME), CD36 is also highly expressed by cancer-associated fibroblasts (CAFs), facilitating FA uptake but also avoiding an excess of lipid peroxidation and ferroptosis [31,32].

Although still unclear, the uptake of exogenous lipids, instead of DNL, plays a predominant role in the proliferation and progression of ICC [33]. Li L et al. identified FATP1 (SLC27A1)-increased expression as a compensatory mechanism to introduce FAs from the TME. Specifically, the study showed that human ICC cells rely on lipid and lipoprotein uptake, and the suppression of FATP1 decreased the in vitro growth of ICC cell lines and enhanced the effect of fatty acid synthase (encoded by FASN) inhibition [11]. Other studies investigated the role of FATP1 in breast and colorectal cancer, where its high expression is associated with a significant decrease in overall survival (OS), and its inhibition in vitro reduced FA uptake and cell viability, suggesting it as a potential therapeutic target [34,35]. In addition, a recent investigation showed a dramatically higher level of FAs in ICC, further supporting the increased fatty acid uptake in this disease. Subsequent mechanistic studies identified FATP5 as the predominant mediator of fatty acid uptake required for ICC growth. Thus, the suppression of FATP5 might represent an innovative therapy against ICC [36].

### 2.2. De Novo Lipogenesis

While dietary sources and lipid synthesis are the major ways to obtain FAs, PLC and several other cancers have been reported to reactivate DNL, making them more independent of exogenous lipids [37]. Moreover, several studies have linked the dysregulation of DNL to MASLD pathogenesis and insulin resistance [21,38,39]. At the biochemical level, DNL is modulated by the activity of different enzymes. As the primary substrate for DNL, glucose undergoes glycolysis to generate pyruvate, which produces citrate via the tricarboxylic acid (TCA) cycle in the mitochondria. Although several studies have suggested that DNL is glucose-derived, thus blocking the availability of glucose which should result in a reduced DNL activity [10,40,41,42], recent studies have drawn attention to fructose and sucrose as substrates of DNL in healthy and fatty liver [43,44]. Once the DNL is activated, citrate in the cytoplasm is converted to acetyl-coenzyme A (CoA) by ATP-citrate lyase (ACLY). Through the activity of acetyl-CoA carboxylase (ACC) 1 and 2 (encoded by ACACA and ACACB), CoA is then converted to malonyl-CoA, which is used to produce fatty acids, and specifically 16-carbon saturated palmitate, through the action of FASN. Subsequently, palmitate can be further elongated into long-chain fatty acids or converted into monounsaturated fatty acids (MUFAs) by the catalytic activity of stearoyl-CoA desaturase (SCD). Finally, synthesized FAs can be used to produce more complex lipids such as triglycerides, phospholipids, and cholesterol.

The process of lipogenesis is regulated primarily at the transcriptional level with the activation of sterol regulatory element-binding proteins (SREBP-1 and SREBP-2) and carbohydrate response element-binding protein (ChREBP) [45,46]. ACLY, ACC, and FASN are rate-limiting enzymes of DNL frequently upregulated in HCC compared to adjacent liver tissue and associated with poor overall survival of HCC patients. A study associated ACLY with the expression levels of stemness-related genes, and its silencing or inhibition affected the migration and invasion of HCC cells. Moreover, ACLY silencing dramatically inhibited the transcriptional activity of β-catenin and decreased the expression of Wnt-responsive genes, suggesting that ACLY could regulate the Wnt/β-catenin cascade in HCC [47]. Another finding reported that non-POU domain-containing octamer binding protein (NONO) promoted HCC progression by enhancing FA biosynthesis by interacting with and increasing ACLY mRNA [48]. It has also been demonstrated that ACLY overexpression upregulated REGγ expression and activated the REGγ-proteasome pathway, leading to changes in the expression of downstream signaling pathway proteins in HCC, thereby promoting cell proliferation, invasion, and migration in vitro as well as tumor growth and metastasis formation in vivo [49]. Another study elucidated ACLY interaction with TME and the prognostic effectiveness of the combined expression of ACLY plus PD1, CTLA4, and potential immune checkpoints in clinical trials, such as a promising strategy [50]. Studying the inhibition of hepatic lipogenesis by the liver-specific knockout of ACC 1 and 2 in mice treated with diethylnitrosamine (DEN) unexpectedly led to an increase in tumor burden compared to controls due to a marked increase in antioxidants, including NADPH and reduced glutathione [51]. This unexpected result was further investigated, and the data obtained showed that the mice lacking both ACC 1 and 2 were the only group with an altered lipogenic phenotype, independent of DEN injection or diet [52]. Also, besides the increased hepatic triglyceride and decreased fat oxidation, the same authors observed that chronic ACC inhibition led to the hyper-acetylation of proteins in the extra-mitochondrial space [53]. The inhibition of ACC also prevented a metabolic switch necessary for hepatic stellate cell (HSC) activation and the ability to impair the profibrogenic activity through TGF-β reduction [54]. Finally, the next step in DNL is the palmitate synthesis catalyzed by FASN. The role of FASN in HCC development depends on the in vivo model used. Specifically, our group demonstrated using hydrodynamic tail vein injection (HTVI) that FASN alone or combined with c-Met, NRAS V12, or SCD1 was insufficient to promote mouse hepatocarcinogenesis [55]. However, FASN is essential for AKT-driven hepatocarcinogenesis through mTOR complex 2 [55]. In another study, our group investigated the role of FASN in a murine HCC model induced by the loss of Pten and the overexpression of c-Met. In this model, the ablation of FASN significantly delayed but did not block hepatocarcinogenesis, and further genomic and lipidomic analysis revealed the upregulation of genes involved in cholesterol biosynthesis, suggesting a functional crosstalk and a novel way to intervene therapeutically [56]. In addition, Glyceronephosphate O-acyltransferase (GNPAT) acetylation stabilizes FASN, avoiding degradation and promoting DNL and tumor development [57]. Interestingly, our group showed that, unlike HCC, ICC tumor cells are less dependent on DNL and more addicted to exogenous FAs [11]. Another study observed that the KDM5C-mediated downregulation of FASN correlated with reduced ICC cell proliferation and invasion [58]. In HCC, the upregulation of SCD1 has been described extensively, revealing its crucial role in cell proliferation and its association with genetic susceptibility to hepatocarcinogenesis in mice [59]. For instance, SCD1 was associated with aberrant palmitate signaling in aggressive HCC and inversely correlated with survival time and directly with tumor recurrence [42]. Also, SCD1 negatively regulated autophagy in human HCC cell lines, determining an adverse prognosis for the patients [60]. However, in a subset of HCCs where SCD1 is inhibited, the upregulation of fatty acid desaturase 2 (FADS2) provided an alternative desaturation pathway explaining metabolic plasticity and suggesting the need to target both fatty acid desaturation pathways to impair HCC growth [61].

### 2.3. Abnormal Fatty Acid Catabolism

Tumor metabolic reprogramming refers to the process by which cancer cells alter their metabolic patterns to support their own growth and proliferation, meeting their energy needs. This phenomenon is recognized as a significant hallmark of cancer. Recently, the importance of lipid metabolic reprogramming, particularly the role of fatty acid oxidation (FAO), has gained attention. Increasing evidence suggests that FAO serves as a crucial source of NADH, NADPH, FADH2, and ATP, linking it to various stages of tumorigenesis, development, and metastasis across multiple cancers, including breast cancer, prostate cancer, glioblastoma, colon cancer, gastric cancer, multiple myeloma, and nasopharyngeal cancer. Furthermore, recent investigations have indicated that FAO can promote tumor resistance by enhancing tumor cell autophagy, improving DNA damage repair, altering apoptosis signaling pathways, and facilitating immune evasion by tumor cells [62,63].

The dysregulation of FA metabolism induced by the metabolic reprogramming of cancer cells has also emerged as a crucial factor in hepatocarcinogenesis. In this regard, FAO is a vital catabolic process that allows cells to use FAs as a source of energy. Also known as β-oxidation, FAO is the mitochondrial process used by the cells to break down long-chain FAs into acetyl-CoA, NADH, and FADH2. Diverse studies linked FAO to tumor growth, metastasis, immune evasion, and chemotherapy resistance [64,65,66]. In PLC, FAO involves enzymes and transporters, such as acyl-CoA oxidases (ACOXs), carnitine palmitoyltransferases (CPTs), medium-chain acyl-CoA dehydrogenase (MCAD), and peroxisome proliferator-activated receptor alpha (PPARα), are often altered in their expression, enabling cancer cells to adapt to their energy demands to sustain survival, proliferation, stemness, drug resistance, and also metastasis [33,64]. A recent study showed that, in an HCC subtype deficient for carbamoyl phosphate synthetase I (CPS1), cells highly depended on FAO to acquire enough ATP for growth rather than using available glucose or glutamine. Furthermore, FAO promoted HCC stemness features through the FOXM1–AKT axis. CPS1 was confirmed as one of the factors inducing metabolic rewiring towards FAO in HCC, and potentially, FAO inhibition might produce a better outcome for patients with CPS1 deficiency [67]. Recently, Senni N et al. [68] showed that oncogenic β-catenin-induced HCCs are not glycolytic but rather oriented towards FAO as an energy source under the control of PPARα and characterized by reduced lipogenesis. The findings of this study may have significant clinical implications because β-catenin gain-of-function mutations are common in HCC development, accounting for 20% to 40% of all HCC cases. Also, it was demonstrated that FAO inhibition or PPARα depletion was sufficient to arrest the initiation and progression of HCC driven by oncogenic β-catenin, indicating that FAO targeting may represent a suitable approach [68]. CPT1 and CPT2 are critical enzymes in the FAO pathway, responsible for delivering long-chain fatty acids to the mitochondria for oxidation, generating ATP and NADPH [69]. Regarding adaptability, the upregulation of these enzymes could provide cancer cells with a metabolic advantage. A recent study revealed that the inhibition of mitochondria fission enhances FAO through the upregulation of CPT1A, inducing proliferation and metastasis, altering the nicotinamide adenine dinucleotide (NAD +)/Sirtuin 1 (SIRT1) axis [70]. The study also demonstrated that an increased mitochondria fission exerted the opposite effects [70]. Another study identified miR-377-3p as a key regulator in the expression of CPT1C and lipid metabolism. Indeed, this miRNA could inhibit CPT1C and consequently suppress β-oxidation, altering HCC growth and invasion in vitro and in vivo [71]. Moreover, it has been shown that both E2F1 and E2F2 can repress CPT2 expression, generating a lipid-rich environment required for MASLD-related HCC development and, consequently, protecting the tumor cells against lipotoxicity [72]. The study uncovered a novel mechanism in which E2F2 is a regulator of lipid metabolism in a manner that is independent of its cell-cycle functions. In addition, CPT2 downregulation promotes FAO low activity and leads to resistance to lipotoxicity cell death by inhibiting the activation of JNK mediated by Src. The subsequent accumulation of acylcarnitine can trigger HCC development by activating STAT3 [73]. Moreover, the downregulation of acylcarnitine translocase (SLC25A20) in the mitochondrial matrix is observed in both HCC and CCA (see TCGA ualcan.path.uab.edu) and has been shown to suppress FAO and promote EMT [74]. In addition, SLC27A5 is associated with FA uptake, and its downregulation elevates TXNRD1 expression via the activation of the KEAP1/NRF2 pathway. The overexpression of SLC27A5 suppresses the growth of hepatoma cells in vitro and in vivo, whilst the blockade of NRF2/TXNRD1 sensitizes SLC27A5-deficient hepatoma cells to sorafenib treatment [75]. Therefore, it seems that FAO supports PLC development and progression, producing FAs useful for structural and messenger molecules instead of storage or energy sources, and this involvement makes it a potential therapeutic target.

## 3. Lipid Signaling

Lipids act as first and second messengers in signal transduction (Table 1). Unlike classical oncogenic signaling pathways centered on protein kinases and transcription factors, the lipid-mediated signaling landscape encompasses a remarkable diversity of bioactive molecules derived from membrane phospholipids, sphingolipids, fatty acids, and cholesterol, creating an intricate communication system that intersects with virtually every aspect of cancer cell biology. Several studies have highlighted the ability of specific lipids, acting as signaling molecules, to interact with oncogenes, altering the signaling pathways and likely promoting carcinogenesis [76,77]. Mounting evidence has negatively associated telomere length with body fat mass. In children with obesity, telomere length is reduced and associated with DNA hypermethylation at the TERT promoter, causing reduced TERT activity compared to control individuals. The study pointed out that erythrocyte lauric acid and total saturated FAs, linoleic acid, and total n-6 PUFAs were higher in obese children [78]. Furthermore, studies involving preclinical murine models upon high-fat diet feeding showed that the shortening of telomeres and telomerase deficiency is associated with hepatocyte metabolic dysfunction, indicating a mechanism for liver injury in the inflamed microenvironment via the activation of the tumor suppressor p53-peroxisome proliferator-activated receptor gamma coactivator 1 alpha (p53-PGC1a) axis [79]. In confirmation of these observations, another study provided insights into the role of microRNA-21 as a possible connection between fatty liver and HCC. Indeed, the authors provide a novel mechanism in which microRNA-21 promotes lipid accumulation in cancer progression by interacting with the Hbp1–p53–Srebp1c pathway [80]. However, TERT expression and its activity are usually restored in HCC, where approximately 80% of all cases show TERT promoter and gene alterations [81]. Moreover, another study investigated the association between obesity and the uptake of free FAs responsible for the induction of EMT programs and HCC development. The study revealed that saturated free FA palmitate-induced EMT in HCC cells by activating the Wnt/β-catenin and TGF-β signaling pathways and, in addition, highlighting the specific role played by CD36 [27]. Sphingolipid metabolism generates potent bioactive molecules, including ceramide, sphingosine, and sphingosine-1-phosphate (S1P), which function as rheostats controlling cell fate decisions between survival and apoptosis [82]. Among sphingolipids, C16-ceramide has been shown to bind and activate the p53 tumor suppressor. The binding stabilizes and disrupts the p53 complex with the MDM2 (mouse double minute 2) E3 ligase, leading to p53 accumulation, nuclear translocation, and activation of the downstream targets [83]. Serum or folate deprivation triggers this mechanism, implying a diverse cellular response to nutrient and metabolic stress. Ceramide was also identified as a second messenger involved in protein translocation into subcellular compartments during transmembrane signal transduction. Specifically, ceramide activates the kinase suppressor of Ras 1 (KSR1), acting as a positive regulator of the RAS–RAF–MAPK pathway, which is frequently altered in liver cancers [84]. Furthermore, another study identified significant alterations of sphingolipid parameters, specifically C16-ceramide and S1P, in the serum of patients with HCC that could potentially be used as diagnostic markers for hepatic diseases [85]. Moreover, the role as a therapeutic target of S1P in liver pathophysiology was investigated in 77 HCC patients who underwent surgical treatment, revealing that S1P levels were usually lower in HCC tissue compared to adjacent tissue, and these levels were associated with poor differentiation and early recurrence [86]. Alternatively, many members of the eicosanoid pathway have pro-inflammatory properties and have been identified as potential treatment targets due to their role in reducing the risk of developing different tumors, including PLC. Eicosanoids (EICs) derived from arachidonic acid metabolism via cyclooxygenase (COX), lipoxygenase (LOX), and cytochrome P450 pathways generate prostaglandins, leukotrienes, and epoxyeicosatrienoic acids, respectively [87]. For example, it is well-known that prostaglandin E2 (PGE2) is a pro-inflammatory lipid mediator that promotes cancer growth. In a recent study, the authors reported that omega-3 polyunsaturated fatty acids (w-3 PUFA) upregulate 15-hydroxyprostaglandin dehydrogenase (15-PGDH) expression by inhibiting miR-26a and miR-26b, thereby contributing to the w-3 PUFA-induced inhibition of human cholangiocarcinoma cell growth, which catalyzes oxidation of the 15(S)-hydroxyl group of PGE2, leading to its inactivation [88]. The significance of the study relies on the induction of 15-PGDH by the use of nontoxic w-3 PUFA to block cholangiocarcinoma growth without inhibiting the antithrombotic prostacyclin PGI2, a cardiovascular side effect associated with COX-2 inhibition. In 2014, a meta-analysis demonstrated that COX-2 expression in HCC was associated with decreased overall and disease-free survival, indicating a worse prognosis [89]. High COX-2 protein has been correlated with the differentiation grade, likely advanced TNM stage, larger tumor size, and increased lymphovascular invasion, suggesting that its abnormal expression plays an important role in hepatocarcinogenesis [90]. The inhibition of COX-2 exhibited anti-tumor activities by preventing the aggressive properties of HCC cells [91]. In contrast with the oncogenic role played by PGE2 and COX-2, prostaglandin D2 (PGD2) is considered an anti-cancer EIC due to its ability to inhibit tumor progression, affecting the self-renewal of cancer stem cells (CSCs) [92]. Instead, the role of LOX is still under debate because of its pro-apoptotic and anti-apoptotic involvement, which has been reported in different cells and tissues [93]. Nosaka T et al. [94] analyzed the contribution of 5-LOX to the progression of HCC. In HCC tissues, they observed a population of CD163+ TAMs that express 5-LOX that start the synthesis of leukotrienes (LTs) LTB4 and LTC/D/E4, thereby enhancing the proliferative and stem cell potential of HCC cells. Moreover, LTB4 has been linked to HBV-mediated HCC development through the activation of ERK signaling. Specifically, LTB4 G-protein-coupled receptors, BLT1 and BLT2, showed opposite functions in promoting/inhibiting tumorigenesis. At the same time, other molecules downstream the EIC pathway, such as group II secretory and cytosolic Phospholipases A2 (sPLA-IIa, cPLA2a), have been associated with HCC promotion and are thereby potential therapeutic targets [87,95,96]. Another study showed through targeted phospholipid analysis that HCC development is characterized by a reprogramming in choline catabolism and phospholipid metabolism [97]. Indeed, the study displayed that choline was significantly increased in tumor tissue, lysophosphatidylcholine (LPC) within bile whilst lysophosphatidic acid (LPA) was increased in the tumor tissue, bile and plasma of HCC patients compared with controls. These alterations could generate a proliferative TME through the paracrine/autocrine signaling activation of G-protein-coupled receptors. Endothelial differentiation gene 2 (EDG2) is a G-protein-coupled LPA receptor that is overexpressed in liver regeneration after liver resection and in HCC tissue compared to normal liver. Indeed, it has been observed that platelets accumulated near remnants of liver tissues during liver resection can release LPA, which has been linked to the enhancement of EMT and thus involved in recurrence and metastasis development [98]. The authors of the study discovered that LPA/EDG2 signaling in an HCC cell line induces an increase in the levels of S-Phase kinase-associated protein 2 (SKP2) with a concomitant downregulation of p27kip1 via upregulating the phosphorylation of AKT and mTOR. Similarly, studies conducted in our laboratory revealed that levels of FASN directly correlate with SKP2 in human HCC specimens. In our study, FASN deletion was paralleled by SKP2 downregulation and p27KIP1 induction in the AKT-driven HCC preclinical mouse model, implying that FASN-positive liver tumors could be targeted with SKP2 inhibitors or p27KIP1 activators [99]. Further investigation on the role of LPA as a signaling molecule showed that this lipid increased MMP-9 expression levels and induced the activation of the p38 mitogen-activated protein kinase (MAPK) signaling. The study demonstrated how LPA induced HCC cell migration, invasion, and adhesion through this mechanism, confirming its potential use as a biomarker and therapeutic target [100]. Conversely, phosphatidylcholine (PC) negatively regulates hepatocarcinogenesis by the induction of death ligands, such as FAS and tumor necrosis factor-alpha (TNF-a), a pathway followed by caspase 8 and 3 activation. The study suggested that PC intake may inhibit HCC development in high-risk patients by enhancing apoptotic signaling [101].

## 4. Fatty Acid Metabolism in Liver Cancer Progression

Recently, it has been demonstrated that lipid signaling not only supports tumorigenesis per se but also plays important roles in cancer progression and metastasis development. A recognized hallmark feature cancer cells exhibit is profound metabolic reprogramming, with alterations in fatty acid metabolism. Several studies have provided better insight into the connections involving FAs and cell migration, angiogenesis, and escape from immunosurveillance.

### 4.1. Regulation of Cell Membrane Structure and Fluidity

The most direct effect of the altered rate of the novo lipogenesis is the change in lipid composition influencing the structure and fluidity of the cell membrane. As a consequence, it has been shown that tumor-associated lipogenesis protects cancer cells, promoting membrane lipid saturation [107]. The elevated rate of DNL in cancer cells facilitates the production of saturated and monounsaturated FAs, providing major stability and being less susceptible to being targeted for peroxidation due to the presence of a few double bonds. This higher degree of saturation at the membrane level improves cell resistance to oxidative stress generated by chemotherapeutic agents [107]. Further, intracellular cholesterol levels can dramatically influence membrane architecture and fluidity, impacting the tumor cell’s ability to become invasive. An increased level of cholesterol is usually related to a pronounced membrane rigidity with the consequence of limiting the capacity of the cells to change their shape and essentially being able to activate the epithelial-to-mesenchymal transition (EMT) program and intra/extravasation from blood vessels necessary for metastatic dissemination. However, some studies showed that increased cholesterol efflux characterizes tumors overexpressing the ATP-binding cassette transporter ABCA1 and displaying higher rates of distant metastases [108]. In addition, rigidity due to increased cholesterol makes the membrane less permeable to anti-cancer agents [109]. Specifically concerning the liver, a study unveiled the deregulation of SCD1 as a mechano-sensitive key enzyme that responds to matrix stiffness and can reprogram lipid metabolism, promoting invasion and metastasis and affecting HCC patients’ survival [110]. Another study pointed out the potential utility of altered lipid metabolism as a diagnostic marker for cancerous cells with the opportunity to treat aggressive HCCs targeting palmitic acid metabolism. The study revealed that treatment with palmitic acid reduced the invasiveness and metastasis formation both in vivo and in vitro by modulating membrane fluidity and limiting glucose availability. At the molecular level, palmitic acid reduced the phosphorylation levels of the mammalian target of rapamycin (mTOR) and signal transducer and activator of transcription 3 (STAT3) pathway proteins [111].

### 4.2. Lipid Metabolism in Tumor Microenvironment and Immunosurveillance

The complexity of the tumor microenvironment (TME) is related to the presence of various cell types, including cancer cells and immune cells (Table 2), fibroblasts, and endothelial cells. Altered lipid signaling and metabolism significantly influence these cells and how they behave, particularly the immune cells, which are crucial for shaping an immunosuppressive environment that enhances tumor progression and poor response to therapy. In this setting, the modulation of lipid signaling influences the recruitment and activation of T cells and dendritic cells, driving their dysfunction and exhaustion, significantly affecting the anti-tumor response to immunotherapy. Indeed, lipid signaling can also alter the response to pro-angiogenic factors such as vascular endothelial growth factor (VEGF), supporting and further enhancing tumor vascularization and facilitating the supply of nutrients and oxygen and the proliferation and migration of tumor cells. In the last decade, immunotherapies, such as immune checkpoint blockade (ICB), have progressed significantly in cancer treatment, although several factors influence its outcome. Several monoclonal antibodies, approved for clinical applications, have been developed to specifically target programmed cell death protein 1 (PD-1) and prevent the interaction with its ligand to enhance the T cell’s anti-tumor effect, thus eliminating tumor cells. However, tumors are characterized by an immunosuppressive TME and abnormal lipid metabolism determining a decreased anti-tumor effect of T cells that is critical in contrasting tumor growth. In this setting, Chen Y et al. developed a lipid metabolism scoring system for patients with lung adenocarcinoma and abnormal lipid metabolism [112]. Combining drug screening, they identified MK1775, which inhibits fatty acid oxidation. The study demonstrates that targeting lipid metabolism with MK1775 can effectively remodel the TME and enhance T cell infiltration, improving anti-PD-1 activity. Recent studies have found that CD36-mediated lipid uptake induces the reprogramming of tumor-associated immune cells, promotes their lipid metabolism, and exerts tumor progression [113]. The uptake of FAs mediated by CD36 affects the anti-tumor immunity of CD8+ T lymphocytes due to intracellular lipid peroxidation and ferroptosis. CD36 can also lead to the excessive accumulation of lipids in macrophages, promoting their differentiation to M2 phenotype TAMs and enhancing tumor progression [114]. Recently, receptor-interacting protein kinase 3 (RIPK3), a central factor in necroptosis, has been identified as the main character of this polarization promoting M2 TAM accumulation in the TME. RIPK3 is often downregulated in HCC–HCC-associated macrophages, significantly suppressing caspase-1-mediated peroxisome proliferator-activated receptor alpha (PPARa) cleavage, thus promoting FAO and increased FA accumulation [115]. Similarly, Liu et al. found that macrophagic S100A4 enhances M2 TAM polarization following the activation of PPARγ-dependent FAO induction [116]. Atezolizumab and bevacizumab are frequently used in combination to treat HCC patients. Atezolizumab is an immune checkpoint inhibitor (ICI) that prevents the interaction between PD-L1 and PD-1, avoiding the immunosuppression of T cells. In contrast, bevacizumab lowers the growth of new blood vessels by inhibiting VEGF-A [117]. Liu et al. have shown that activating fatty acid-binding protein 5 (FABP5) limited FAO and induced lipid accumulation in monocytes/macrophages. The activation of FABP5 is negatively related to HCC patients’ survival due to the expression of PD-L1 on Treg cells induced by an increase in IL-10 via the Jnk/Stat3 pathway. Their results highlight the role of FABP5 in fostering immune tolerance acquisition and the potential role as a therapeutic target for both tumor-associated monocytes (TAMs) and cancer cells [118]. Another multi-omics approach demonstrated the efficacy of combining atezolizumab and bevacizumab in patients with hepatic steatosis. Mechanistically, lipid accumulation in HCC patients induced an increment in PD-L1 levels, promoting an immunosuppressive environment. However, the steatotic HCCs were susceptible to combined immunotherapy, suggesting that intratumoral lipid accumulation might be an imaging biomarker to predict ICI’s efficacy [119]. Indeed, a previous study revealed that immunosuppressive functions characterize Tregs and M2 macrophages through manipulating cellular metabolism toward FAO to obtain energy, delineating a potential strategy based on targeting altered metabolism associated with immunotherapy [120]. In an additional investigation, Chen et al. discovered that patients with CTNNB1 mutation display a reduction in activated immune cells, suggesting that CTNNB1 mutations might represent a potential biomarker for better stratification of HCC patients who could benefit from ICI treatment [121]. These data corroborate the study of Senni et al., which shows how FAO is the primary energy provider of CTNNB1-mutated HCCs through the transcription factor PPARa. Pharmacologic or genetic inhibition could provide a therapeutic strategy to treat CTNNB1-mutated HCC patients [68]. Schmidt N et al. identified acyl-CoA:cholesterol acyltransferase (ACAT) as a direct anti-carcinogenic. This study showed that ACAT inhibition in HBV-related HCC reduces CD8+ T cells, altering lipid composition and enhancing TCR signaling and TCR-independent bioenergetics. The results of this study suggest that ACAT inhibition potentially rescues the T cells from high-cholesterol environments, boosting their capacity to enhance PD-1 blockade response [122]. Recently, the natural killer (NK) T cells have emerged as major immune modulators in tumor immunity. NKs are CD1d T cell populations with both adaptative and innate features that mostly recognize lipid antigens to execute their immune response by producing and secreting large amounts of cytokines. Altered lipid metabolism and signaling will affect the immunomodulatory function of these cells [123]. Tang W et al., using a high-fat and high-carbohydrate diet in MASLD-HCC mouse models and transcriptome analysis on the human liver, observed that aberrant cholesterol metabolism suppressed NK immunosurveillance. In this study, the authors highlighted the mTORC1/SREBP2/cholesterol axis as responsible for NK dysfunction in the context of MASLD liver microenvironment and provided strategies to reactivate NKs to control obesity-related HCC [124]. Similarly, Ringel A et al. have investigated how obesity impairs CD8+ T cell function in the murine TME, promoting tumor growth [125]. The metabolic shift in TME due to the upregulated pathways that mobilize free FAs in response to the HF diet might also be responsible for the altered functionality of PD-1+ CD8+ T cells, weakening the response to ICIs [126]. In another study, Cheng X et al., using transcriptomic and lipidomic analysis, observed that aberrant lipid metabolism induces the senescence of invariant NK (iNK) cells, weakening their immune surveillance capacity and anti-tumor potential. Specifically, the senescence status was promoted by the accumulation of long-chain acylcarnitines (LCACs) in HCC tissue, especially palmitoyl-carnitine and stearoyl-carnitine impairing the function and expansion of iNKs [127].

### 4.3. Lipid Metabolism and Regulation of Cancer Epigenome

Evidence suggests that FA metabolism is a critical regulator of the liver cancer epigenome, fostering malignant transformation through complex metabolic rewiring and consequent epigenetic modifications. Indeed, disrupted FA homeostasis contributes to the accumulation of metabolites that serve as signaling molecules, substrates, cofactors, and inhibitors for epigenetic modifications or reshaping the chromatin landscape, thereby establishing a metabolic–epigenetic axis that promotes carcinogenesis [134,135]. A central role for ACLY and acyl-CoA synthetase short-chain family member 2 (ACSS2) has been described, as these enzymes are the primary source of acetyl-CoA, a fundamental substrate for histone acetylation, thereby establishing a direct link between FA synthesis and histone modification that influences chromatin accessibility and gene expression [136,137]. The lysine acetylation of histones is sensitive to acetyl-CoA availability and is balanced by histone acetyltransferases (HATs) and histone deacetylases (HDACs). AKT signaling controls histone acetylation through the modulation of ACLY and acetyl-CoA synthesis. Alterations in this mechanism lead to the hyper-acetylation of histones and subsequent activation of oncogenic programs, including those controlled by SREBPs and ChREBP, key transcription factors governing lipid and carbohydrate metabolism [138,139]. A recent study revealed that, upon ER stress, Sec63 activation stabilizes ACLY, thus increasing the supply of acetyl-CoA and lipid biosynthesis. Moreover, Sec63 entered the nucleus to actively coordinate with ACLY for the expression of Snail1 via epigenetic modification. Finally, Sec63 promoted HCC metastasis, and a clinically high expression of Sec63 predicted an unfavorable prognosis of HCC patients [140]. Further, acetyl-CoA facilitates ketone body synthesis like β-Hydroxybutyrate (β-HB) in the liver. Β-HB is an endogenous and specific inhibitor of class I HDAC, and its upregulation led to the identification of a new histone modification, lysine β-hydroxybutyrylation (Kbhb). Importantly, this modification upregulated a set of distinct genes with different functions from others that bear lysine acetylation and methylation [141,142]. FAO influences the NAD+/NADH ratio and consequently modulates the activity of NAD+-dependent deacetylases such as sirtuins [143]. Specifically, SIRT1 and SIRT3 function as metabolic sensors, translating changes in fatty acid utilization into epigenetic modifications, and their inhibition in the context of metabolic stress contributes to the aberrant acetylation of histones, promoting genomic instability and oncogenic transformation [144].

In the last few years, new treatment strategies have included the so-called “epidrugs”, which essentially inhibit chromatin readers and modifiers to prevent PLCs by altering epigenetic modifications. For example, the study by Jühling et al. [145] reveals that chronic hepatitis C (CHC) and MASH share epigenetic and transcriptional changes associated with HCC risk in patients, and that risk is reduced by the use of a small inhibitor targeting the chromatin reader Bromodomain 4 (BRD4). The authors show that liver disease induced by epigenetic alterations is a target for HCC chemoprevention, and that reverting these alterations can effectively reduce cancer risk in patients by restoring the transcriptional reprogramming of genes. However, these epidrugs show intrinsic limitations, which are related to high toxicities and the development of drug resistance.

On the other hand, numerous efforts have been made to improve the Clustered Regularly Interspaced Short Palindromic Repeats/CRISPR-associated protein 9 (CRISPR/Cas9) system for the identification and validation of HCC gene functions and carcinogenesis mechanisms. CRISPR/Cas9 adapted for epigenetic editing represents an emerging technology for reactivating genes with high selectivity, enabling alternative options in HCC clinical management. Moreover, epigenetic editing technology has been combined with single-cell sequencing and machine learning to explore its utility further and expand its application in HCC pathogenesis [146]. In a recent study performed by Sgro et al. [147], the authors bioinformatically analyzed a panel of 12 tumor suppressor genes (BCO2, CDKN2A, CPS1, HHIP, miR-122-5p, MT1E, MT1M, PSAT1, PTGR1, PZP, TMEM106A, and TTC36) that are epigenetically silenced and under-expressed in HCC tumor samples compared to normal tissue. Using CRISPR-activation (CRISPRa) systems, they demonstrate the ability to reactivate some of these genes in cell lines and confirm the superior locus selectivity of gRNA systems compared to epigenetic drugs, such as decitabine and vorinostat.

### 4.4. The Lipolytic Pathway and Lipid Droplets Accumulation

Instead of DNL as the main source of FAs, recent studies have highlighted that certain cancer cells use the lipolytic pathway to produce free FAs from stored lipid to support tumor growth [148,149]. Lipolysis is a catabolic pathway that sequentially hydrolyzes triglycerides stored in lipid droplets into free fatty acids (FFAs) and glycerol, primarily mediated by adipose triglyceride lipase (ATGL), hormone-sensitive lipase (HSL), and monoglyceride lipase (MGLL). This process is tightly regulated by hormonal signals that activate protein kinase A (PKA) via cAMP, leading to the phosphorylation and translocation of HSL to lipid droplets (LDs), thereby enhancing triglyceride breakdown and mobilizing FFAs for β-oxidation in mitochondria or exporting to peripheral tissues [150]. In the liver, lipolysis not only provides energy substrates during fasting or stress but also influences systemic lipid homeostasis by coupling with fatty acid uptake proteins such as FAT/CD36, which facilitate FFA internalization following depalmitoylation. Therefore, in primary liver cancer, lipolysis emerges as a critical player in tumor progression by reprogramming lipid metabolism to support rapid proliferation, membrane biogenesis, and survival in nutrient-scarce microenvironments. HCC cells exhibit enhanced intracellular lipolysis, which alters the expression of enzymes such as MGLL hydrolyzes monoacylglycerols into free FAs and glycerol, releasing FAs from stored lipids that fuel de novo lipogenesis, migration, and signaling pathways, underscoring a lipolytic–lipogenic axis that sustains tumorigenesis despite the availability of exogenous lipids [151]. This metabolic flexibility allows cancer cells to utilize stored triglycerides via lipophagy, an autophagic process that degrades LDs by lysosomal acid [152]. Of note is that MGLL is under transcriptional control of the Yap protooncogene. Indeed, it has been shown that promoter methylation of large tumor suppressor kinase 1 (LATS1) results in dysfunction of the Hippo signaling pathway, which enhances dephosphorylation and nuclear transport of YAP, inducing the overexpression of MGLL in HCC [151]. Aberrant lipolysis also converges with hypoxia and acidosis in the tumor stroma, promoting metastasis by altering FA desaturation through regulators such as Raf-1. Overall, lipolysis’s dual role as an energy source and a lipid signaling molecule makes it a promising target for HCC therapies, with preclinical evidence linking its dysregulation to a poor prognosis [153]. Preclinical studies demonstrated that ATGL inhibitors induced toxic FA accumulation, triggering lipotoxicity, ER stress, and apoptosis in liver cancer models. Similarly, targeting lysosomal acid lipase (LAL) in lipophagy impairs FA hydrolysis, inducing LD buildup and suppressing tumor progression, especially in advanced HCC [152]. The activation of the liver X receptor alpha (LXRα) and the inhibition of a Raf-1-SCD1 protein complex cause the intracellular accumulation of saturated free fatty acids, leading to lethal lipotoxicity in tumor cells via oxidative stress and PERK/CHOP-mediated apoptosis, and are effective in MASH-driven HCC mouse models [154].

LDs consist of neutral lipids such as TAGs, cholesteryl esters, and retinyl esters, acting as storage organelles for lipid and energy homeostasis [155]. Physiologically, LDs also have a protective role by sequestering potential toxic lipids, thereby preventing any unregulated lipolysis or lipid peroxidation and halting processes such as ferroptosis or any cytotoxic effect [155,156]. In this perspective, the protective effect played by LDs in cancer cells may facilitate a compensatory high antioxidant activity due to increased reactive oxygen species (ROS) generation, contributing to cancer cell survival and growth. A study on breast cancer reported how cancer cells use LDs for cell survival mechanisms against ROS-mediated nutrient and lipotoxic stress, highlighting a potential therapeutic strategy involving the inhibition of TAG synthesis, LD formation and the promotion of lipolysis [157]. Concerning PLC, a study identified BNIP3, a mitochondrial cargo receptor, as an HCC cell growth suppressor by accelerating LD turnover at the lysosome in a manner dependent on BNIP3-binding LC3 [158]. On the other hand, long-chain acyl CoA synthetase 4 (ACSL4) modulates DNL by accumulating intracellular triglycerides and cholesterols, and promoting LD buildup, furthering the progression of HCC [159]. Furthermore, different studies revealed that HCC and other tumor cells, when exposed to hypoxia, accumulate LDs by stimulating and increasing the expression of Lipin 1 via HIF-1. Lipin 1 upregulation enables ER and redox homeostasis during oxygen deprivation, supplying FAs from LDs for mitochondrial energy production and promoting cell proliferation [160]. There is a clear need to expand research on LDs in the context of PLC, focusing on elucidating the mechanisms behind their accumulation to identify new targets and develop novel treatments.

## 5. Targeting Lipid Metabolism in Liver Cancer Treatment

Given the extensive role played by FAs in cancer pathogenesis, targeting lipid metabolism and signaling has raised clinical interest as a promising approach to develop new therapeutic ways that could alleviate the burden of chronic liver diseases and treat cancer. Considering that lipid metabolic rewiring has the potential to function as a tumor-promoting factor and the ability to induce therapy resistance, a plethora of different molecules with inhibitory activity have been designed that interact at different sites and levels of lipid metabolism, targeting de novo FA synthesis, FA oxidation, and exogenous lipid uptake. Table 3 overviews lipid metabolism targets and substances tested in cancers, specifically primary liver cancer. Specifically concerning primary liver cancer, many preclinical studies have focused on targeting de novo lipogenesis enzymes. For instance, the ACC inhibitor ND-654, which mimics the effects of ACC phosphorylation, was found to block DNL and the development of HCC. When administered alone at a dose of 10 mg/kg/day, and in combination with sorafenib, also at 10 mg/kg/day, ND-654 improved the survival rates of tumor-bearing rats. In this study, the combination of both compounds was particularly effective, reducing the incidence of HCC by 81% compared to the control group. Additionally, HCC progression was linked to the dysregulation of AMPK-mediated ACC phosphorylation, highlighting the potential of ACC inhibitors as a treatment option for liver cancer [161]. Other molecules targeting ACC, such as MK-4074 [162] and GS-0976 (Firsocostat) [163,164], have been developed mainly for patients presenting with MASH (metabolic dysfunction-associated steatohepatitis, ex NASH) or MASLD. However, treatment with these ACC inhibitors was associated with plasma hypertriglyceridemia due to the activation of SREBP-1c and increased VLDL secretion. Furthermore, some clinical trials have been conducted to evaluate the combination of ACC and diacylglycerol acyltransferase 2 (DGAT2) inhibition in reducing hepatic steatosis in early clinical trials [165,166]. Regarding ACLY, several synthetic inhibitors have been developed. Among them, ETC-1002 or Bempedoic acid have reached phase III in clinical trials evaluating their long-term efficacy in patients with hyperlipidemia and at high cardiovascular risk and who are statin-intolerant [167,168,169]. ETC-1002 exerts its pharmacological effects primarily in the liver, where it is converted to its active form, ETC-1002-CoA, by acyl-CoA synthase. In preclinical mouse models, ETC-1002-CoA inhibits ACLY, completely disrupting DNL by attenuating hepatotoxin DEN and high-fat diet-induced hepatocellular carcinogenesis. Moreover, it increases AMPK activity, affecting the phosphorylation of ACC and HMG-CoA reductase [170,171]. Since the discovery of FASN as an oncogenic target, most efforts have been focused on its inhibition, blocking proliferation, and inducing the apoptosis of cancer cells. Among the several inhibitors developed, C75 [99,172] and cerulenin [173] are the first generation and were extensively tested in liver cancer treatment. However, despite their significant anti-tumor effects in preclinical models, they failed due to limited pharmacokinetic properties, severe side effects, and tissue distribution [174]. Orlistat is an FDA-approved pancreatic lipase inhibitor designed to control obesity. Moreover, it has been used in in vitro and in vivo studies as a potent inhibitor of FASN [175,176]. Since 2014, several compounds, such as GSK2194069, TVB-3166, TVB-2640, Fasnall, JNJ-54302833, IPI-9119, and FT113, have been developed to achieve highly potent reversible or irreversible FASN inhibition [177]. Among them, only TVB-2640 (Denifanstat) have reached clinical trials to date. TVB-2640 has demonstrated promising results in phase I/II trials for solid tumors, including non-small cell lung cancer (NSCLC), breast cancer, and astrocytoma. Its anti-tumor activity was demonstrated both as a single agent and in combination with paclitaxel, exerting its anti-tumor activity, modulating the TME, and improving the response to immunotherapy. Moreover, it exhibited a manageable safety profile with nonserious adverse events [178]. Concerning the liver, in the FASCINATE-2 trial, a phase II study, TVB-2640 represented a candidate drug that could substantially improve the treatment of MASH patients [179,180]. The results observed in this trial, for both MASH resolution and fibrosis regression, led to further testing in phase III trials. However, as reported on clinicaltrials.gov, FASCINATE-3 trial ID NCT06594523 and FASCINIT trial ID NCT06692283 have been withdrawn for business decision. In HCC, FASN inhibition with orlistat or TVB-2640 reduced the palmitoylation of MHC-I, avoiding its lysosomal degradation. Specifically concerning cholangiocarcinoma treatment, sphingolipid metabolism has been targeted; to be precise, the enzyme sphingosine kinase 2 with the inhibitor ABC294640 showed promising results in vitro, in vivo and in clinical trials [181,182,183,184]. However, as reported on clinicaltrials.gov, a phase II study ID NCT02939807 for patients with advanced HCC who have experienced tumor progression or unacceptable toxicity on single agent sorafenib has been withdrawn for being rewritten for different disease populations. In addition, some inhibitors in HCC treatment target the stearoyl-CoA Desaturase-1 (SCD-1) pathway that can also regulate the tumor sensitivity to sorafenib [185]. Furthermore, targeting FAO through CPT-1 inhibition with etomoxir, despite successfully reducing HCC occurrence in preclinical models, was abandoned due to hepatotoxicity [186,187]. Finally, several statins, such as atorvastatin, simvastatin, and pravastatin, alone or in combination therapy, are under investigation for patients with HCC [188,189].

## 6. Serum Lipid Profile and Lipidomic Tools Studying Liver Cancer

In recent decades, numerous studies have sought to determine the relationship between serum lipid profiles and PLC. Blood cholesterol metabolism is often disrupted in patients with PLC, especially those with HCC. Research has shown that these patients frequently exhibit paradoxically lower levels of total cholesterol and LDL compared to healthy individuals. This phenomenon may result from impaired hepatic synthesis and altered lipoprotein metabolism due to compromised liver function. Moreover, the HDL-to-LDL ratio, which is commonly used as a marker for cardiovascular risk, demonstrates altered patterns in HCC patients. Some studies suggest that lower cholesterol levels may correlate with poorer prognosis and a more advanced disease stage. TG levels can vary depending on the extent of liver damage and underlying conditions such as cirrhosis or hepatitis. A meta-analysis involving a population of 10,765,221 participants, of whom 31,055 had liver cancer, indicated that serum total cholesterol, TG, and HDL cholesterol levels are negatively associated with liver cancer risk. This suggests that higher concentrations of these lipids may be linked to a reduced risk of liver cancer. However, no significant relationship has been found between LDL cholesterol levels and liver cancer risk [190,191]. Another study, which involved a long-term follow-up of ICC patients who underwent surgical resection, confirmed that HDL cholesterol is an independent predictor of overall survival, recurrence-free survival, and early recurrence. These findings underscore the importance of monitoring and managing HDL cholesterol levels in these patients, as low HDL levels may indicate a poor prognosis [192]. Similar results emerged from a study of patients with MASLD, which suggested that HDL cholesterol levels could serve as a novel predictive marker for HCC and its aggressiveness. Low HDL cholesterol, along with increased waist circumference and altered metabolic pathways, are predisposing factors for these patients. This emphasizes the need to integrate clinical approaches with healthier lifestyle choices to prevent the development of HCC [193,194]. Additionally, emerging evidence from clinical trials indicates that cholesterol-lowering statins may have protective effects against the development of HCC, although the underlying mechanisms are still under investigation.

**Figure 4:**
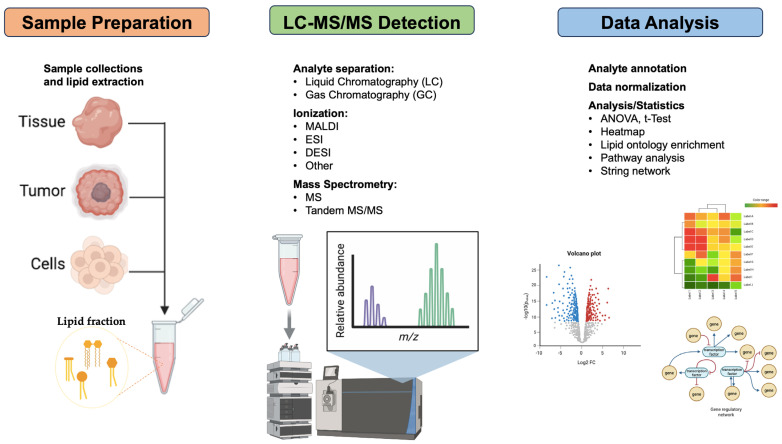
Schematic lipidomic workflow for primary liver cancer research. Three main steps to lipidomic analysis including sample preparation, MS detection, and data analysis. Abbreviations: ESI, electrospray ionization; MALDI, matrix-assisted laser desorption ionization; DESI, desorption electrospray ionization; MS, mass spectrometry; ANOVA, analysis of variance. This image was modified using the BioRender online tool (www.biorender.com, accessed on 10 November 2025).

To gain a better understanding, lipidomics arises as the comprehensive analysis of lipid species within biological systems. Since its introduction in 2003, it has emerged as a powerful approach for investigating the complex roles of lipids in cancer biology. Similar to other omic-based techniques, lipidomics provides critical insights into disease mechanisms, therapeutic target development, and diagnostic biomarkers identification. Modern lipidomics tools integrate sophisticated analytical technologies, computational methods, and systems biology approaches to characterize lipid metabolism and signaling alterations that contribute to malignant transformation, progression, and resistance to therapy (Figure 4). In lipidomics, three mass spectrometry (MS) methods are mainly used: direct injection shotgun MS, MS combined with chromatographic separation, and mass spectrometric imaging (MSI) [195]. As omics technologies continue to evolve, their integration into cancer research and the help of fast-growing artificial intelligence (AI) promises to unlock new dimensions in our understanding of tumor biology, leading to the development, in this scenario, of new approaches based on lipid-targeted precision oncology (Table 4).

Some studies have already pointed out the importance of FA composition in hepatic tissue and circulating as well as the imbalance in their ratios as the liver progresses towards HCC [196,197,198]. Several saturated FAs and MUFAs are increased during the progression from chronic hepatitis to cirrhosis to HCC. Specifically, the levels of MUFAs (16:1) and (18:1) progressively rise in the setting of viral-associated HCC [199]. Conversely, serum levels of PUFAs associated with diacylglycerol (DAG) and triacylglycerol (TAG) are decreased in the blood of patients with HCC [200,201,202]. Patterson et al. investigated the aberrant lipid metabolism in HCC through plasma metabolomics and lipid profiling. They found that the plasma from HCC patients was enriched in glycodeoxycholate, deoxycholate 3-sulfate, and bilirubin and also presented an upregulation of biliverdin and some other fetal bile acids. Moreover, lignoceric acid and nervonic acid, two very long-chain fatty acids (VLCFAs), were remarkably decreased in the plasma of HCC subjects compared to cirrhosis and healthy controls [203]. However, whether or not these VLCFAs have a role in HCC progression is to be debated. Accordingly, VLCFAs as lipid mediators and the very long-chain fatty acid elongase (ELOVL) family are involved in hepatocarcinogenesis [204,205]. In another study, proteomic and lipidomic profiling was used on Pten-null mice to investigate MASH liver, tumors, and circulating fatty acid composition. The relevance to human MASH and HCC was further validated. The results obtained highlighted the role played by lipid-modifying enzymes converting saturated FAs to MUFAs in HCC and the importance of an increased ratio of long-chain n6-PUFAs over n3-PUFAs associated with MASH and HCC risk [199]. Morita et al. performed imaging mass spectrometry revealing the increase in phosphatidylcholine (PC) species with palmitoleic acid or oleic acid at the sn-2-position and the reduction in lysophosphatidylcholine (LPC) with palmitic acid at the sn-1-position in HCC tissues. LPCAT1, which catalyzes the conversion of LPC to PC, was upregulated and responsible for cell proliferation, migration, and invasion [206]. Collectively, these studies and many others show that lipidomics contribute to a better understanding of dysregulation of lipid metabolism and signaling in HCC, especially when combined with transcriptional studies, providing the potential discovery of new biomarkers for disease diagnosis and progression.

## 7. Conclusions

The therapeutic targeting of fatty acid metabolism in primary liver cancer presents a notable paradox. Despite a strong mechanistic understanding, clinical translation has stalled, revealing flaws in current drug development strategies. Metabolic adaptation, now recognized as a hallmark of cancer, allows malignant cells to survive in hostile environments, with lipid dysregulation playing a complex role in cancer progression. Lipids serve not only as energy sources but also as essential components of cell membranes, intracellular signaling molecules, and modulators of the tumor microenvironment and immune response. As the body’s central metabolic hub, the liver is particularly vulnerable to lipid-driven carcinogenesis. PLC, a leading cause of cancer-related mortality worldwide, shows significant metabolic reprogramming characterized by changes in fatty acid uptake, de novo lipogenesis, fatty acid oxidation, and lipoprotein secretion. These processes are closely linked to specific oncogenes and related signaling pathways, including Wnt/β-catenin and MYC-dependent molecular cascades. Subsequent phase III studies targeting lipogenesis (Fascinate-3 and FASCINIT) were withdrawn for “business reasons,” likely due to the competition from GLP-1 agonists rather than scientific failures. This situation highlights a strategic error: developing fatty acid metabolism inhibitors primarily for MASH endpoints when the biology of HCC presents a more compelling rationale, especially in the context of immunotherapy synergies.

The critical challenge in targeting cancer cells lies in their remarkable metabolic flexibility and the activation of compensatory pathways. For instance, when SCD1 is inhibited, FADS2 can compensate for the disruption. Similarly, if FASN is removed/inactivated, the biosynthesis of cholesterol is upregulated. In cases of ICC where de novo lipogenesis is inhibited, the cancer cells can switch to exogenous lipid uptake through FATP1 and/or FATP5. This metabolic plasticity, along with the interconnectedness of glycolysis, mitochondrial respiration, and glutamine metabolism, means that targeting single enzymes or pathways often fails to produce satisfactory therapeutic responses. Additional complications arise from significant tumor heterogeneity; for example, HCC and ICC have fundamentally different lipid dependencies. HCC driven by AKT relies on DNL, while β-catenin-mutated HCC depends on FAO. Moreover, hepatotoxicity presents paradoxes, such as when inhibiting ACC, which can lead to hypertriglyceridemia, or when inhibiting CPT1, which can be directly toxic to the liver. Therapeutic strategies must also consider dietary lipid sources that can bypass biosynthetic blockades and recognize the dual nature of lipid accumulation. Lipids can be pro-tumorigenic through signaling pathways but can also have anti-tumorigenic effects due to lipotoxicity.

Presumably, the most promising yet underutilized opportunity in cancer treatment lies in combining FA metabolism inhibitors with immunotherapy. Several mechanisms support this approach. For instance, CD36-mediated FA uptake can lead to ferroptosis and exhaustion in CD8+ T cells. Additionally, M2-like TAMs rely on fatty acid oxidation (FAO) through PPARα/PPARγ pathways to maintain their immunosuppressive characteristics. The inhibition of FASN reduces the palmitoylation of MHC-I, which enhances surface presentation and tumor recognition. Moreover, the activation of FABP5 promotes the development of PD-L1+ regulatory T cells via IL-10/JNK/STAT3 signaling. Despite this evidence, there are currently no clinical trials that combine FA metabolism inhibitors with immune checkpoint inhibitors. The observation that the combination of atezolizumab and bevacizumab shows increased efficacy in steatotic HCC suggests that intratumoral lipid accumulation could serve as an imaging biomarker for patient stratification; however, this approach is absent from current trial designs.

Recent advancements in lipidomics and metabolomics have transformed our understanding of lipid signatures associated with specific oncogenic events and metabolic states. When combined with gene expression data and mass spectrometry imaging, these technologies allow for precise mapping of lipid signaling organization, which can serve as biomarkers for early detection and insights into malignant signaling networks. To move forward, we need to shift away from the monotherapy paradigm towards rational combination trials that address metabolic plasticity and exploit immune–metabolic interactions. We should also focus on selecting patients based on their lipid dependency signatures. Immediate opportunities include repurposing existing drugs, designing biomarker-driven patient stratification using multi-omics approaches, and prioritizing combinations of immunotherapy. Ultimately, we need to strategically reorient our efforts from single-target development to precision combination approaches that reflect the biological complexity of lipid metabolism in liver cancer.

## Figures and Tables

**Table 1:** Lipid mediators and fatty acid derivatives.

Mediator	Primary Functions	Pro/Anti-Inflammatory	Angiogenesis & Proliferation Immunosuppression	References
PGE2 (Prostaglandin E2)	Vasodilation, pain sensitization, fever induction, mucus secretion	Pro-inflammatory (early stages); Anti-inflammatory (inhibits T cell proliferation, promotes Treg)	Pro-angiogenic via VEGF upregulation; Pro-proliferative in cancer cells; Inhibits T cell activation, promotes Treg differentiation, suppresses NK cells	[87]
PGD2 (Prostaglandin D2)	Sleep regulation, vasodilation, mast cell mediator	Pro-inflammatory—recruits Th2 cells, eosinophils, basophils	Anti-proliferative in many cancer types; Immunomodulatory, promoting Th2 responses	[87,92]
PGI2 (Prostacyclin)	Vasodilation, platelet inhibition	Anti-inflammatory—inhibits leukocyte adhesion, reduces cytokine production	Pro-angiogenic, promoting endothelial cell migration; Proliferation context-dependent in cancer; Reduces T cell activation	[87]
TXA2 (Thromboxane A2)	Platelet aggregation, vasoconstriction	Pro-inflammatory—promotes leukocyte adhesion, platelet activation	May promote tumor angiogenesis; Pro-proliferative in some cancers	[102]
LTB4 (Leukotriene B4)	Neutrophil chemotaxis, leukocyte activation	Strongly pro-inflammatory, recruits neutrophils, enhances vascular permeability	Pro-angiogenic—recruits endothelial progenitors, promotes neovascularization; Pro-proliferative in various cancer cell lines via BLT receptors	[93,94]
LXA4 (Lipoxin A4)	Resolution of inflammation, anti-neutrophil actions	Anti-inflammatory/Pro-resolution—stops neutrophil recruitment, promotes macrophage efferocytosis	Promotes physiological angiogenesis, inhibits pathological neovascularization; Anti-proliferative in cancer cells; Promotes Treg function, inhibits DC maturation	[103,104]
LXB4 (Lipoxin B4)	Resolution of inflammation	Anti-inflammatory Pro-resolution	Promotes physiological angiogenesis, inhibits pathological neovascularization; Immunosuppressive activity	[105]
PAF (Platelet-Activating Factor)	Platelet activation, neutrophil priming	Strongly pro-inflammatory, systemic inflammation, anaphylaxis, septic shock	Pro-angiogenic; Pro-proliferative in various cell types; Immune-activating	[106]
S1P (Sphingosine-1-phosphate)	Lymphocyte trafficking, vascular integrity, cell survival	Can be pro- or anti-inflammatory depending on receptor	Strongly pro-angiogenic, promoting endothelial cell migration, survival; Pro-proliferative via survival and proliferation signaling; Immunosuppressive, sequesters lymphocytes in lymph nodes	[82]
Ceramide	Apoptosis, cell senescence, stress responses	Pro-inflammatory	Anti-angiogenic; Anti-proliferative; Pro-apoptotic	[82,83]
LPA (Lysophosphatidic acid)	Cell proliferation, migration, survival	Pro-inflammatory, recruits immune cells, activates platelets	Pro-angiogenic, stimulating endothelial cell migration; Strongly pro-proliferative, mitogenic in many cell types; Low immunosuppression	[87,95,96]
LPC (Lysophosphatidylcholine)	Membrane remodeling, inflammation	Pro-inflammatory—oxidized form particularly inflammatory	Pro-angiogenic in atherosclerosis	[97]

**Table 2:** Fatty acid metabolism of immune cells in the PLC TME.

Immune Cells	Primary Function	Mechanism/Consequence	Effect on Tumor	References
M2-like TAMs	Promote immunosuppression, angiogenesis, tissue remodeling, and metastasis	FAO increased (CPT1A); Lipid droplets accumulation; Increased lipid uptake via CD36 Immunosuppressive cytokines (IL10)	Activated/polarized to pro-tumor M2; Growth support; Promotes anti-inflammatory phenotype; Inhibits T cell responses	[128,129]
MDSCs	Suppress T and NK cell function, promote Treg induction, and drive tumor progression	Lipid uptake through arginase-1/iNOS increased; FAO increased via CD36/CPT1A; T cell suppression	Immune evasion; Activated/accumulation of PMN-MDSC, M-MDSC; Abundant in HCC, especially with hepatic steatosis	[128,129]
Tregs	Suppress anti-tumor immunity and maintain immune homeostasis	Increased FA uptake, mitochondrial FAO maintenance and immunosuppression	Immunosuppressive; FAO sustains suppressive function; Promotes tumor immune evasion; Inhibits effector T cells; Enriched in lipid-rich HCC; Adaptation to TME, CD8 inhibition	[128,130]
CD8+ T cells	Anti-tumor cytotoxicity	Lipid dysregulation (cholesterol/FA), FAO disruption, peroxidation with loss/dysfunction; decreased IFN-γ/IL-2; Reduced infiltration	Impaired anti-tumor function; Reduced proliferation and cytotoxicity; Exhaustion phenotype; Decreased IFN-γ production; Poor infiltration into tumor	[128,131]
CD4+ T cells	Anti-tumor surveillance	Suppressed FAO in lipid-rich TME; Metabolic reprogramming toward lipid storage; Selective depletion in NAFLD-HCC; Impaired anti-tumor response	Impaired anti-tumor function; Decreased IFN-γ production; Reduced help for CTLs; Functional exhaustion	[128,131]
Natural Killer (NK) Cells	Cytotoxicity against tumor and produce inflammatory cytokines (IFN-γ)	Lipid metabolites suppress cytotoxicity in tumor-prone liver	Anti-tumor reduced: Impaired infiltration/killing reducing immune surveillance	[128]
DCs	Antigen presentation	Lipid accumulation impairs maturation/antigen presentation; Impaired FAO leads to mitochondrial dysfunction	Anti-tumor weakened: Poor T cell priming; Fatty TME prevents DC maturation and migration to lymph nodes	[132,133]

**Table 3:** Fatty acid inhibitors used in preclinical and clinical research.

Inhibitor	Mechanism of Action	Observed Effects	References	Clinical Trial ID/Status
ND-654 ND-646	ACC inhibitor	Reduced tumor growth and DNL	[161]	Not in clinical trials
MK-4074	ACC inhibitor	Reduced DNL	[162]	
Firsocostat (GS-0976)	ACC inhibitor	Reduced DNL	[163,164,165,166]	NCT02891408/C+R NCT02856555/C+R NCT03987074/C+R NCT04971785/C+R NCT02781584/C+R
ETC-1002	ACLY inhibitor	Block DNL	[167,168,169,170,171]	NCT02988115/C+R NCT02666664/C+R NCT02991118/C+R NCT03001076/C+R NCT02988115/C+R
C75	FASN inhibitor	Induced apoptosis, reduced cell growth	[99,172]	Not in clinical trials
Cerulenin	FASN inhibitor	Induced apoptosis	[173]	Not in clinical trials
Orlistat (Xenical)	Pancreatic lipase inhibitor, FASN inhibitor	Reduced cell proliferation, induced apoptosis	[175,176]	FDA-approved for controlling obesity
GSK2194069 TVB-3166 Fasnall JNJ-54302833 IPI-9119 FT113	FASN inhibitors	Reduced fatty acid synthesis	[177]	Not in clinical trials
TVB-2640 (Denifanstat)	Selective FASN inhibitor	Reduced tumor growth, decreased palmitate synthesis	[178,179,180]	NCT02223247/C NCT04906421/C+R NCT06594523/W NCT06692283/W
ABC294640	Sphingosine kinase 2 inhibitor	Induced autophagy	[181,182,183,184]	NCT03377179/C NCT02939807/W
MF-438 A939572	SCD1 inhibitor	Reduced cell growth, induced ER stress	[185]	Not in clinical trials
Etomoxir ST1326	CPT1 inhibitor	Reduced fatty acid oxidation, induced apoptosis	[186,187]	Not in clinical trials
Atorvastatin simvastatin pravastatin	HMG-CoA reductase inhibitor	Under investigation	[188,189]	NCT03024684/A+NR NCT02968810/A+NR NCT04133792/A+NR NCT01418729/C NCT01903694/C

Status clinical trial: A+NR, active not recruiting; C, completed; C+R, completed with results; W, withdrawn.

**Table 4:** Lipidomic techniques for liver cancer research.

Technique	Characteristics	Advantages	Limitations	Applications
Mass Spectrometry (MS)	High sensitivity; Can be coupled with separation techniques; Identifies lipids based on mass-to-charge ratio	Comprehensive lipid profiling; High accuracy and resolution; Can detect thousands of lipid species	Expensive instrumentation; Complex data analysis; Ionization efficiency varies between lipid classes	Biomarker discovery; Profiling tumor vs. non-tumor tissue; Investigating drug resistance mechanisms; Monitoring lipid changes during hepatocarcinogenesis
Liquid Chromatography-MS (LC-MS)	Combines chromatographic separation with MS detection	Separates isomeric and isobaric species; Improves lipid identification; Better for complex samples	Longer analysis time; Requires optimization of LC conditions	Large-scale lipid profiling; Clinical sample analysis
Shotgun Lipidomics	Direct infusion into MS without prior separation; Uses MS/MS for lipid identification	Rapid analysis; High throughput; Suitable for large sample sets; Simplified workflow	Ion suppression effects; Lower sensitivity for low-abundance lipids; Limited structural information	Screening lipid profiles in clinical samples; High-throughput biomarker discovery; Comparative studies of large patient cohorts
Imaging Mass Spectrometry	Spatial distribution of lipids in tissue; Can use MALDI or DESI ionization; Maintains tissue architecture information	Preserves spatial information; Can visualize heterogeneity within tumor; No extraction needed; Links lipids to histopathology	Limited sensitivity compared to LC-MS; Lower lipid coverage; Resolution limitations; Sample preparation is critical	Mapping tumor margins based on lipid profiles; Visualizing lipid distribution in TME; Correlating lipid changes with histopathological features; Studying tumor heterogeneity
Nuclear Magnetic Resonance (NMR)	Non-destructive analysis; Based on magnetic properties of atomic nuclei; Provides structural information	Minimal sample preparation; Highly reproducible; Quantitative without standards; Non-destructive	Lower sensitivity than MS; Limited dynamic range; Spectral overlap challenges	Metabolic profiling of liver tissues; Monitoring lipid changes during disease progression; Investigating membrane fluidity alterations; Lipid metabolism studies
Raman Spectroscopy	Label-free vibrational spectroscopy; Can be used for imaging; Based on inelastic light scattering	Non-destructive and label-free; Minimal sample preparation; Can be used in vivo; Good for spatial analysis	Lower sensitivity; Limited lipid class specificity; Background fluorescence interference; Less quantitative than MS	Real-time imaging of lipids in liver tissues; Studying lipid droplet accumulation in HCC; Monitoring treatment response; Intraoperative tissue assessment
Fluorescence Microscopy	Uses lipid-specific fluorescent dyes; High spatial resolution; Can be combined with other staining methods	Excellent for subcellular localization; Compatible with live cell imaging; Can track dynamic processes	Limited to targeted lipid classes; Potential dye artifacts; Limited quantification	Visualizing lipid droplets in liver cancer cells; Tracking lipid trafficking in tumor cells; Studying lipid raft alterations; Investigating lipid–protein interactions
Lipidomic Flux Analysis	Uses stable isotope labeling; Tracks metabolic conversion of lipids	Provides dynamic information; Reveals altered lipid metabolism	Complex data analysis; Requires specialized software; Limited to in vitro/ex vivo	Investigating altered lipid synthesis pathways in HCC; Studying fatty acid oxidation in liver cancer; Elucidating lipid remodeling mechanisms

## Data Availability

Data sharing is not applicable. No new data were created or analyzed in this study.

## Abbreviations

ABCA1, ATP-binding cassette transporter; ACACA, Acetyl-CoA carboxylase alpha; ACACB, Acetyl-CoA carboxylase beta; ACAT, Acyl-CoA:cholesterol acyltransferase; ACC, Acetyl-CoA carboxylase; ACLY, ATP-citrate lyase; ACOX, Acyl-CoA oxidases; ACSS2, Acyl-CoA synthetase short-chain family member 2; AI, Artificial intelligence; AKT, AKT Serine/Threonine Kinase; AMPK, AMP-activated protein kinase; AP-1, Activating protein-1; ATP, Adenosine triphosphate; BLT, Leukotriene B4 receptor; CAF, Cancer-associated fibroblast; CD36, Cluster of differentiation 36; CEBPβ, CCAAT Enhancer binding protein beta; ChREBP, carbohydrate response element-binding protein; CoA, Coenzyme A; COX, Cyclooxygenase; CPS1, Carbamoyl phosphate synthetase I; CPT, Carnitine palmitoyltransferase; CSC, Cancer stem cell; CTLA4, Cytotoxic T-lymphocyte associated protein 4; CTNNB1, Catenin Beta 1; DAG, Diacylglycerol; DEN, Diethylnitrosamine; DGAT2, Diacylglycerol acyltransferase 2; DNL, De novo lipogenesis; EDG2, Endothelial differentiation gene 2; EIC, Eicosanoid; ELOVL, Elongation of very long-chain fatty acids protein; EMT, Epithelial–mesenchymal transition; ER, Endoplasmic reticulum; ERK, Extracellular signal-regulated kinases; FA, Fatty acid; FABP, Fatty acid-binding protein; FADH2, flavin adenine dinucleotide; FADS2, Fatty acid desaturase 2; FAO, Fatty acid oxidation; FAS, Fas cell surface death receptor; FASN, Fatty acid synthase; FAT, Fatty acid translocase; FATP, Fatty acid transport protein family; FOXM1, Forkhead box M1; GCO, Global Cancer Observatory; GNPAT, Glyceronephosphate O-acyltransferase; HAT Histone acetyl transferase; β-HB, β-Hydroxybutyrate; HBV, Hepatitis B virus; HCC, Hepatocellular carcinoma; HCV, Hepatitis C virus; HDAC, Histone deacetylase; HIF, Hypoxia-inducible factor; HMG-CoA reductase, 3-Hydroxy-3-methylglutaryl-CoA reductase; HSC, Hepatic stellate cell; HTVI, Hydrodynamic tail vein injection; ICB, Immuno checkpoint blockade; ICC, Intrahepatic cholangiocarcinoma; IL-10, Interleukin 10; JNK, C-Jun N-terminal kinase; KBHB, lysine β-hydroxybutyrylation; KDM5C, Lysine Demethylase 5C; KEAP1, Kelch like ECH associated protein 1; KSR1, Kinase suppressor of Ras 1; LCAC, Long-chain acylcarnitines; LDL, low-density lipoprotein; LOX, Lipoxygenase; LPA, Lysophosphatidic acid; LPC, Lysophosphatidylcholine; LPCAT, Lysophosphatidylcholine acyltransferase; LT, Leukotriene; MASH, Metabolic dysfunction-associated steatohepatitis; MASLD, Metabolic dysfunction-associated steatotic liver disease; MAPK, Mitogen-activated protein kinase; MCAD, Medium-chain acyl-CoA dehydrogenase; MDM2, Mouse double minute 2; MHC-I, Major histocompatibility complex, class I; MMP-9, Matrix metalloproteinase 9; MS, Mass spectrometry; MSI, Mass spectrometric imaging; mTOR, Mechanistic target of rapamycin kinase; MUFA, Monounsaturated fatty acid; NADPH, Nicotinamide adenine dinucleotide phosphate; NAFLD, non-alcoholic fatty liver disease; NASH, Non-alcoholic Steatohepatitis; NF-κB, Nuclear Factor Kappa B; NK, Natural killer; NOGO-B, Neurite outgrowth inhibitor; NONO, Non-POU domain-containing octamer binding protein; NRAS, Neuroblastoma RAS viral oncogene homolog; NRF2, Nuclear factor erythroid 2-related factor 2; NSCLC, Non-small cell lung cancer; PC, Phosphatidylcholine; PD1, Programmed cell death 1; PD-L1, Programmed cell death 1 ligand 1; PGC1a, Peroxisome proliferator-activated receptor gamma coactivator 1-alpha; 15-PGDH, 15-Hydroxyprostaglandin dehydrogenase; PGE_2_, Prostaglandin E2; PI3K, Phosphoinositide 3-kinase; PLC, Primary liver cancer; PPARα, Peroxisome proliferator-activated receptor alpha; PSC, Primary sclerosing cholangitis; Pten, Phosphatase and tensin homolog; PUFA, polyunsaturated fatty acid; REGγ, 11S Regulator complex subunit gamma; RIPK3 Receptor-interacting protein kinase 3; S1P, Sphingosine-1-phosphate; SCD, Stearoyl-CoA desaturase; SIRT1, Sirtuin 1; SKP2, S-Phase kinase-associated protein 2; SLC27, Solute carrier 27 protein family; SREBP, Sterol regulatory element-binding protein; STAT, Signal transducer and activator of transcription; TAG, Triacylglycerol; TAM, Tumor-associated macrophage; TCA, Tricarboxylic acid; TCR, T cell receptor; TERT, Telomerase reverse transcriptase; TGF-β, Transforming growth factor beta; TME, Tumor microenvironment; TNF-a, Tumor necrosis factor-alpha; TXNRD1, Thioredoxin reductase 1; VEGF, Vascular endothelial growth factor; VLCFA, Very long-chain fatty acid; VLDL, Very low-density lipoprotein.
